# Molecular Detection of *Plasmodium* Infection among Anophelinae Mosquitoes and Differentiation of Biological Forms of *Anopheles Stephensi* Collected from Malarious Areas of Afghanistan and Iran

**DOI:** 10.4314/ejhs.v32i2.7

**Published:** 2022-03

**Authors:** Vahideh Moin-Vaziri, Navid Dinparast Djadid, Helen Hoosh-Deghati, Hoda Atta, Abbas Ali Raz, Seyyed Javad Seyyed-Tabaei, Naseh Maleki-Ravasan, Sedigheh Zakeri

**Affiliations:** 1 Department of Parasitology, School of Medicine, Shahid Beheshti University of Medical Sciences, Tehran, Iran; 2 Malaria and Vector Research Group, Biotechnology Research Center, Pasteur Institute of Iran, Tehran, Iran; 3 Malaria Control, Word Health Organization/Eastern Mediterranean Regional Office, Cairo, Egypt; 4 Department of parasitology, Pasteur Institute of Iran, Tehran, Iran

**Keywords:** Anopheles stephensi, Plasmodium spp, OBP1 gene, Iran, Afghanistan

## Abstract

**Background:**

Updated information on the vectorial capacity of vectors is required in each malarious areas as well in Iran and its neighboring countries such as Afghanistan. The aims of this study were to investigate the potential infection of about 800 specimens collected from malarious areas of Afghanistan and Iran, and to differentiate biological forms of Anopheles stephensi.

**Method:**

Two molecular markers, 18S RNA gene subunit and AsteObp1 intron I, were used respectively for investigation Plasmodium infection and identifying the biological forms of An. stephensi.

**Results:**

Plasmodium infection was detected in 4 pools of Afghanistan specimens, including An. stephensi, collected from Nangarhar. Individually examination showed infection in 5 An. stephensi (infection rate: 1.25), to P. falciparum (2), P. vivax (2) and a mix infection. Out of five infected specimens, three were intermediate forms and two were mysorensis. No infection was found in specimens collected from Iran (Chabahar County), probably due to the active malaria control program in south-east of Iran.

**Conclusion:**

The key role of An. stephensi, as a known Asian malaria vector, was re-emphasized in Afghanistan by the results achieved here. The fauna of vectors and the pattern of biological forms of An. stephensi are similar in both countries that urge regional investigations to provide evidence-based and applied data for decision-maker in malaria control.

## Introduction

Malaria is a life-threatening vector borne disease which caused by five protozoan organisms; *Plasmodium falciparum, P. vivax, P. malariae, P. ovale*, and most recently *P. knowlesi* (1, 2). The incidence rate of malaria has declined globally; in 2018 an estimated 228 million cases of malaria occurred worldwide, compared with 251 million cases in 2010 and 231 million cases in 2017. Global investment in malaria control and elimination efforts have also diminished from an estimated US$ 2.7 billion in 2018 compared to US$ 3.2 billion in 2017 (WHO 2019) (3, (4). The malaria elimination network is widening, with more countries moving towards zero indigenous cases: in 2018, 49 countries reported fewer than 10 000 cases, up from 46 countries in 2017 and 40 countries in 2010 (WHO 2019). Iran is targeting malaria elimination by 2020. Disease trends in Iran have declined from 1847 to 81 cases between 2010 and 2016. According to the last report of WHO, zero indigenous cases reported in Iran in 2018 and 2019, with interventions mainly focusing on the detecting malaria cases, managing malaria foci and the imported malaria cases, vector control, and preventing onward transmissions (3,5,6).

One of the problematic issues in malaria elimination in Iran is neighboring with malarious areas of Pakistan and Afghanistan (both countries are in the control phase of malaria) (4) and unfortunately facing with population movement which could highly effect on malaria situation (4). Further, these countries have almost the same profile of vectors and parasites as Iran. In the south east of Iran, *Anopheles fluviatilis, An. dthali, An. stephensi, An.culicifacies* and *An. superpictus* are regarded as malaria vectors (7–9). Nearly the same vector species has been reported for Afghanistan with a minor difference; *An. hyrcanus* instead of *An. dthali* is regarded vector in Afghanistan (10). Accordingly, it seems essential to conduct regional investigations on different aspects like composition of malaria vectors, their possible *Plasmodium* infection and genetic variation of *Plasmodium*. Previously, an inventory research was done on the composition of *Anopheles* species of selected malarious areas of Afghanistan and Iran by the same authors (11), which resulted in identifying *An. superpictus, An. stephensi* & *An. hyrcanus* among 400 specimens collected from Badakhshan, Herat, Kunduz, Nangarhar provinces provided by WHO/EMRO through a joint regional collaboration. Alongside *An. stephensi, An. fluviatilis, An. culicifacies* and *An. sergenti* was reported among 400 specimens collected from Chabahar county of Iran (11). All of them are among the known vectors of malaria and all of them were reported among the checklist of mosquito fauna in both countries (12,13).

Since vector-borne diseases (VBDs) are dynamic and very complicated, basic research on the vectorial capacity of vectors is of great importance (14). The goal of the present study was to investigate the potential *Plasmodium* infection of the specimens collected from the mentioned areas. For decades, parasitological method was the most common method for detection of *Plasmodium* spp. in the salivary glands of *Anopheles* mosquitoes. Direct microscopy method is highly accepted, but it has some shortcomings like being a time-consuming method and heavily dependent on the microscopist skills. In addition, parasite detection would be impossible due to the degeneration of the parasite by passing time. Also, the risk of the false-negative results exist in case of low parasitaemia (15). DNA-dependent methods are common for detection of pathogens in the vectors. These techniques are more sensitive and provide more accurate results (15). On the other hand, different *Anopheles* species, even at population level, have a different ecology, biology, and vectorial capacity (14). Molecular methods could also be a powerful tool for identification of closely related species and different biological forms of the vectors (16). *Anopheles stephensi*, a known vector of malaria, has three different biological forms called *An. stephensi* type, *An. stephensi* mysorensis and *An. stephensi* intermediate (17, 18) which had shown different vectorial capacities and biological characteristics. Another goal of the current study was to detect the biological forms of *An. stephensi* collected in two countries.

## Material and Methods

**Mosquito collection**: About 400 specimens of adults *Anopheles* mosquitoes were previously collected from malarious areas of Afghanistan (Badakhshan, Kunduz, Nangarhar, and Herat) through WHO/EMRO coordination and 400 specimens from Iran (Chabahar county), all of them were identified at species level using reliable keys by expert medical entomologist (19). Details of the study areas and sampling sites are available in the previous published article (11).

**DNA extraction**: Genomic DNA of each specimen was extracted using Cinna pure DNA kit (Cinaclone, Iran, Cat. No.: PR881613) based on the manufacturer's instructions. In brief, 100 µl of Pre-lysis buffer and 10 µl Ributinas were added to each microtube containing the sample, then homogenized and put at 55 °C for 1 hour. After incubation, 400 µl of Lysis buffer was added to each tube and vortexed for 20 sec. Then 300 µl of Precipitation solution was added and vortexed for 5 sec. The solution was transferred to a spin column with collection tube; the tubes centrifuged at (12100 x g, 13000 rpm) for 1 min. The spin column was placed in a new collection tube and 400 µl of Wash buffer was added, centrifuged again at the same situation. Flow-through was discarded and precipitant was washed again with 400 µl of Wash buffer and centrifugation was done as before for 1 min. The column was carefully transferred to a new 1.5 ml tube. Then 50 µl of preheated elution buffer was placed in the center of the column, the lid was closed and incubated for 3–5 min at 65°C. Thereafter, centrifugation was done at 12100 x g (13000 rpm) for 1 min to elute the DNA. Extracted DNA was stored at 4°C until use.

**Nested-PCR protocol for parasite detection**: The goal of the Nested-PCR was to amplify species-specific sequences of the small sub-unit ribosomal ribonucleic acid (ssrRNA) genes of *Plasmodium* species. The first run of PCR was done by *Plasmodium* genus-specific primers (rPLU5: (5′-CCTGTTGCCTTAAACTTC-3′)), (rPLU6: (5′-TTAAAATTGTTGCAGTTAAAACG-3′)); in case of any amplification, the second run was carried out by species-specific primers (rFAL1,2-rVIV1,2- rMAL1,2), ) to identify *Plasmodium* species. PCR assay was carried out basically according to Pinheirob et al (20). The first run amplified about 1200 bp of *Plasmodium* genome, and in the second run the expected band for *P. malariae, P. falciparum* and *P. vivax* was 145, 205, and 120 bp respectively. PCR production was visualized using 1.5% agarose gel electrophoresis. To save time and reduce the cost, pooling method was utilized as follows; an equal portion (1 µl) of five extracted DNA templates was pooled and 10 µl of pooled DNA was used as template. In case of any *Plasmodium* infection, the DNA of each specimen in the infected pool was examined individually.

**PCR protocol for identifying *Anopheles stephensi* biological forms**: To be sure about the morphologically identifies specifies, rDNA-ITS2-PCR was used for species identification of infected specimens. Also, to identify the biological forms of *An. stephensi*, the specific primers named OBP1F (5′-CGTAGGTGGAATATAGGTGG-3′) and OBP1R1A (5′- TCGGCGTAACCATATTTGC-3′) were used, they target the Odorant Binding Protein 1 (*Asteobp1*) (21). PCR reaction was performed in a total of 20 µl. The reaction mixture contained 1 µl of each specific Obp1 primers, 0.2 µl Taq polymerase, 8 µl of master mix, 3 µl of DNA template and 6.8 D.D.W. The amplification profile was as follows: initial denaturation 94 °C for 5 min, followed by 35 cycles of denaturation at 94°C for 30 sec, annealing at 60 °C for 1 min, extension at 72 °C for 50 sec with an additional 10-min extension time in 72 °C in the last cycle. A product of about 845 bp was expected for all biological forms. The differentiation was done by sequencing PCR products.

**Analysis**: PCR product was purified by agarosegel fractionation, using the Perfect Prep Gel Cleanup (Eppendorf, Germany). Direct sequencing of DNA strands was performed by Qiagen (Hilden, Germany) using the primers used for DNA amplification. The sequences were double checked with Chromas Software version 2.31 (www.technelysium.com.au/chromas.html) and manually adjusted, if necessary. The sequences were edited via the CLUSTALW software package (www.ebi.ac.uk/clustalw) and compared with the sequences deposited in the GenBank using BLAST. Phylogenetic tree was constructed using (MEGA5) software version 5.1.

**Ethics Statements**: The project was approved by the committee on the ethics of research department, School of Medicine, Shahid Beheshti University of Medical Sciences, under Permit Number: IR.SBMU.SM.REC.1394.89. Informed consent was not used as human participants were not involved in this project.

## Results

The authors had access to some *Anopheles* specimens collected from selected malarious areas of Afghanistan and Iran, with the morphological outcomes published previously (11). Here, we only present the results of *Plasmodium* infection of collected specimens, as well the biological forms of infected *Anopheles stephensi*.

**Detection of *Plasmodium* spp in collected *Anopheles* spp using Nested-PCR**: *Plasmodium* infection screening was performed in 800 Anophelinae specimens distributed in 160 pools. None of the 400 specimens (80 pools) collected from Iran (Chabahar County) were positive for human malaria *Plasmodium*.

**Figure 1:**
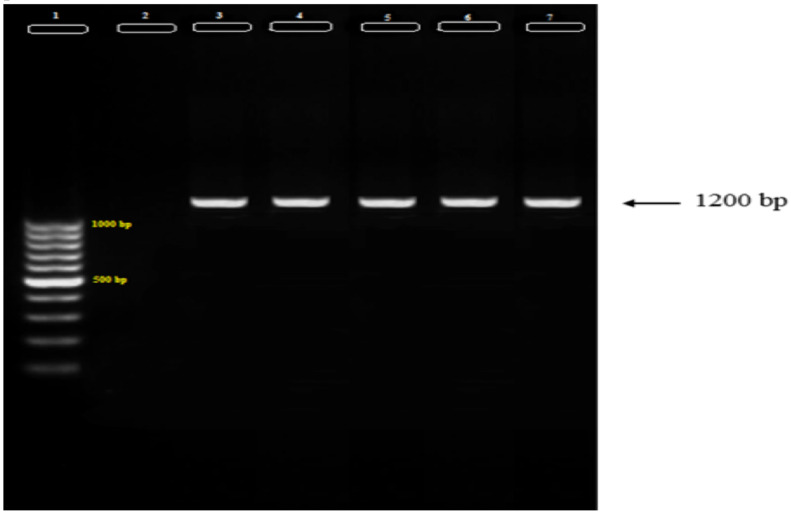
Amplification products obtained in first run of Plasmodium ssrRNA18-Nested-PCR in infected pools of Anopheles stephensi collected from Nangerhar, Afghanistan **Lane 1**: Marker, SinaClone DNA ladder 100 bp, Ready to use **Lane 2**: Negative control; Lane 3: Positive control; Lane 4–7: Positive pools (current study)

However, PCR amplification of 18ssrRNA in 400 specimens (80 pools) collected from Afghanistan showed *Plasmodium* infection in 4 pools (MIR = 5%) by amplification of a band of about 1200 bp (Figure 1).

These pools consisted of *Anopheles stephensi* and were collected from Nangarhar. Identification of *Plasmodium* spp. was performed by a second run of Nested-PCR. Each pool consisted of 5 specimens, in order to assess the infection rate, *Plasmodium* infection of all specimens in infected pools was evaluated individually by the same method. Totally, *Plasmodium* infection was found in 5 *Anopheles stephensi* indicating 1.25 infection rates among 400 collected samples of Afghanistan.

**Figure 2:**
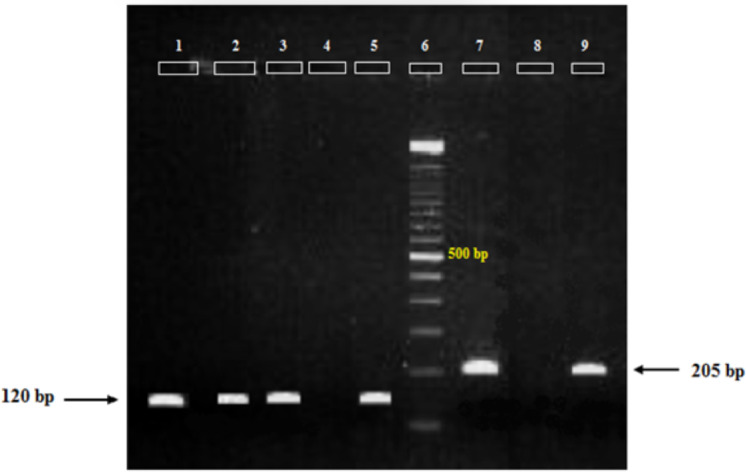
Amplification products obtained in second run of Plasmodium ssrRNA18-Nested-PCR in infected pools of Anopheles stephensi collected from Nangerhar, Afghanistan **Lane 1–3**: P. vivax (current study); Lane 4,8: Negative control, Lane 5: Positive control for P. vivax, Lane 6: Marker, SinaClon DNA ladder 100 bp, Ready to use, Lane 7: Positive control for P. falciparum; Lane 9: P. falciparum (current study)

Based on the second run of PCR the infection was observed to *P. falciparum* (a band of about 205 bp), *P. vivax* (a band of about 120 bp), and mix infection (both two bands) in 2, 2 and 1 samples (Figure 2).

**Molecular identification of *Anopheles stephensi***: The amplified fragment in all specimens was 650 bp; species-specific band for *An. stephensi*. The match between sequence data here and NCBI data confirmed that all specimens are *An. stephensi*.

**Figure 3:**
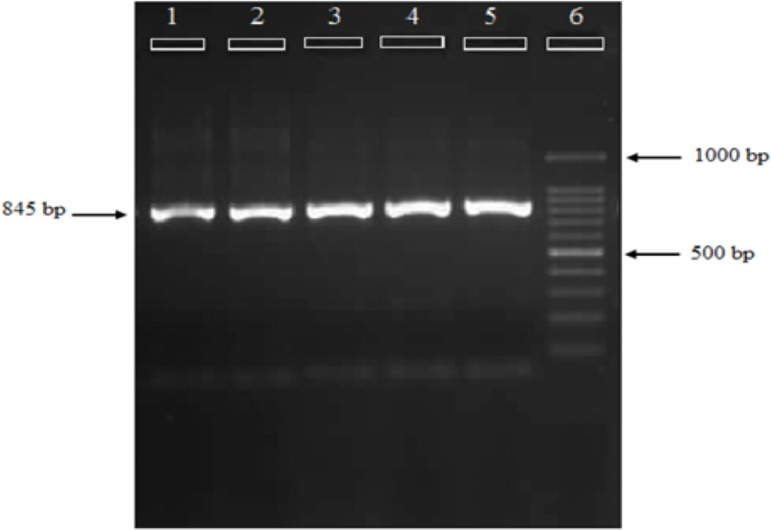
Agarose gel electrophoresis of Odorant Binding Protein 1 (Asteobp1) gene in infected Anopheles stephensi collected from Afghanistan. Lane 1–5: current study samples; Lane 6: SinaClone molecular weight marker (100 bp DNA Ladder)

**Identification of the biological forms of infected *Anopheles stephensi* and molecular analysis**: In order to recognize the biological forms of infected *An. stephensi*, Odorant Binding Protein 1 gene was amplified. It showed a single band of about 845 bp in all biological forms of *An. stephensi* (Figure 3), it was submitted in GenBank (Accession Numbers: KT587049 to KT587053). Comparative *Asteobp1* gene sequences of 5 *An. stephensi* individuals showed 31 polymorphic sites within 845 sequenced nucleotides (3.7%), from which 28 (87.88%) was substitution and 4 (12.12%) due to deletion.

**Figure 4:**
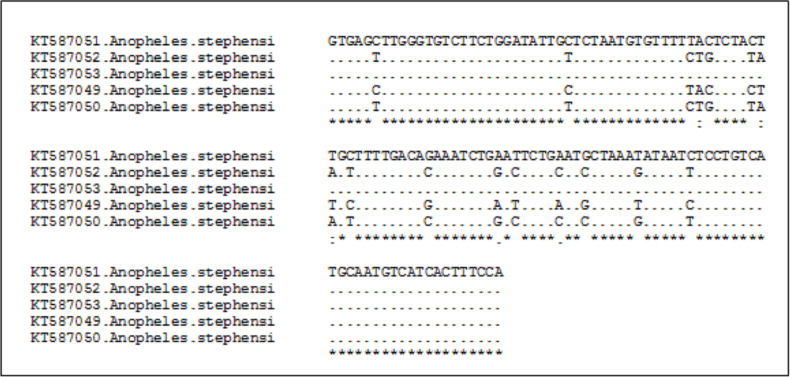
Comparison of nucleotide characters of intron I sequences of Anopheles stephensi collected from Afghanistan (similar sequences showed by dash) (−)

As intron I sequences of *An. stephensi* obp1gene (*Ansteobp1* Intron I), with a length of about 120 bp, was introduced as a powerful marker for differentiating biological forms most of the analysis was done on this part. Totally, 16 polymorphic sites (13.33%) were observed across the sequence nucleotide of *Ansteobp1* Intron I (Figure 4) of five infected *An. stephensi*. Comparison with sequences available in GenBank showed 99% similarity between three specimens and the intermediate form (Accession No: KT587050, KT587052, KT587053), two others showing 99% similarity with the mysorensis form (Accession No: KT587049, KT587051).

**Figure 5:**
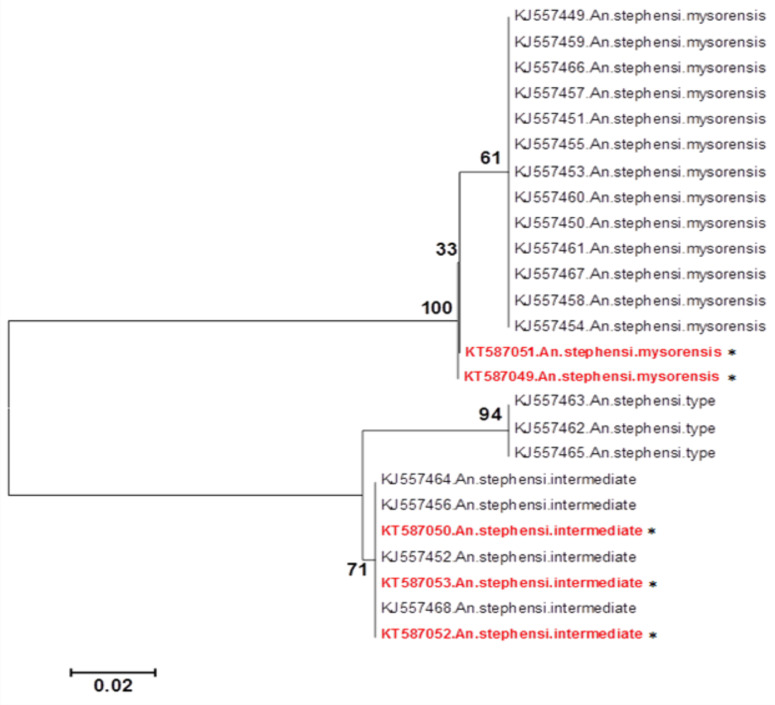
Phylogenetic tree representing the relationship of Ansteobp1 Intron I sequences from five Anopheles stephensi collected from Nangerhar, Afghanistan (Accession No: KT587050, KT587052, KT587053: intermediate form) and (Accession No: KT587049, KT587051 Mysorensis form) and different biological forms of An. stephensi retrieved from GenBank

A Phylogenetic tree was constructed based on the *Ansteobp1* Intron I sequences of 5 infected *An. stephensi* of the current study and some samples of different biological forms of *An. stephensi* retrieved from GenBank (Figure 5). It well shows that three specimens (Accession No: KT587050, KT587052, KT587053) belong to the intermediate form and two others (Accession No: KT587049, KT587051) are mysorensis form supporting the result of BLAST.

## Discussion

Although the malaria global reports has shown a descending trend, but this parasitic disease which infect red blood cells, killed roughly 445 000 in 2016 worldwide (22)(20). Totally, 21 countries, including Iran, were identified by WHO as having the potential to eliminate malaria by 2020 (23). According to this report, the snapshot of indigenous malaria cases in Iran were 57 in 2017 which in comparison to 81 reported cases in 2016, showed a notable decline. It should be mentioned that Iran had zero indigenous cases in 2018 and 2019 (23). Vector control is a key preventive strategy for malaria elimination, so updated data on the vector species profile even at the population level seems necessary. Bordering with Pakistan and Afghanistan has always its effect on malaria in Iran urging regional contribution, both in administrative and research aspects. This study aimed to investigate the potential infection of Anophelinae species to *Plasmodium* parasite collected from selected malarious areas of Afghanistan and Iran.

Although all identified species (*An. stephensi, An. fluviatilis and An. culicifacies*) from Chabahar County of Iran, were considered as powerful malaria vectors (24, 25), no *Plasmodium* infection was detected. This was not far from expectation, most probably due to the proper control strategy and low malaria prevalence, which was also reported by other researchers recently (16). Iran has a border of about 1845 km with Pakistan and Afghanistan in the eastern part of the country with illegal migration and population movement especially from Afghanistan due to the civil wars. Mobile populations and migrant workers are always regarded as a key factor in the spread, care, control and treatment of malaria in the border areas (6, 26, 27), which motivate regional research.

The majority of malaria cases (95%) in Afghanistan are due to *P. falciparum* and 5% to *P. vivax*. Almost 90% of confirmed cases of *P. falciparum* and *P. vivax* in 2017 were reported from 6 provinces including Nangarhar, Laghman, Kunar, Nuristan, Khost and Paktika. Out of 10 malaria deaths, 7 occurred in Nangarhar, 2 in Kabul and 1 in Kunar (28). Among the specimens collected from Afghanistan, *Plasmodium* infection was detected only in five *An. stephensi* captured from Nangarhar as a high-risk area in Afghanistan. Considering the malaria situation in Nangarhar, the obtained results sound reasonable.

*An. stephensi*, as a driving species in the history of malaria transmission is among known malaria vectors in Persian Gulf areas as well in Iran and Afghanistan (9, 16, 24–27, 29, 30). This species is also regarded as an important urban vector in other Asian countries including India, Pakistan, the Arab Peninsula, Iraq, Syria, etc. (31–33). Also, the first report of the presence of this Asian malaria vector and its possible role in the resurgence of malaria in Africa was announced in 2014 by Faulde et al. (34)

*An. stephensi* has three different biological forms; Type, Intermediate, and Mysorensis (18), with distinctly different biological features, transmission capacity and urbanization tendencies. *An. stephensi* type is urban while the mysorensis and intermediate forms are found in rural areas.(9, 18, 35, 36). Type form of *An. stephensi* mainly breeds in wells, cisterns, roof gutters, barrels, buckets, and ornamental tanks. However, the forms in rural areas breed in streams and channels, tanks and ponds, water seepages, and irrigation wells (37). These three forms have similar morphology in all life cycle except in the eggs morphology (ridge number) (18). So in adults, the molecular markers could be useful for their differentiation. Intron I sequences of *An. stephensi* Obp1 were introduced as a proper molecular marker to differentiate the biological forms (21). Based on the *Asteobp1*sequence, two different biological forms, mysorensis and intermediate, were identified among infected *An. stephensi* collected from Nangarhar. Although *An. stephensi* type form was considered as a more important vector especially in India (38, 39), the intermediate form was also introduced as an efficient vector in some other malarious areas such as in Fars province and in the southern part of Iran, in the absence of any type form (35, 36). Further, it was shown that, *An. stephensi* mysorensis is susceptible to *Plasmodium vivax* VK210B (40). It seems that the profile of vectorial capacity of different biological forms of *An. stephensi* in Iran and Afghanistan is more similar to each other than India (40). Absence of infection in *An. stephensi* collected from Chabahr County may be due to the success and achievement of ongoing active malaria control program in the region, as being in the malaria elimination phase. The results obtained in Afghanistan re-emphasize the key role of *An. stephensi* in malaria transmission. The findings of this study can be useful for decision-makers in the study areas.
